# A 3D Microfluidic Model to Recapitulate Cancer Cell Migration and Invasion

**DOI:** 10.3390/bioengineering5020029

**Published:** 2018-04-08

**Authors:** Yi-Chin Toh, Anju Raja, Hanry Yu, Danny van Noort

**Affiliations:** 1 Department of Biomedical Engineering, 4 Engineering Drive, National University of Singapore, Singapore 117853, Singapore; biety@nus.edu.sg; 2 Institute of Bioengineering and Nanotechnology, A*STAR, The Nanos, #04-01, 31 Biopolis Way, Singapore 138669, Singapore; 3 Integrated Health Information Systems (IHiS), 6 Serangoon North Avenue 5, Singapore 554910, Singapore; anju.mythreyi.raja@ihis.com.sg; 4 Department of Physiology, Yong Loo Lin School of Medicine, MD9-04-11, 2 Medical Drive, Singapore 117597, Singapore; medyuh@nus.edu.sg; 5 Mechanobiology Institute, National University of Singapore, T-Lab, #05-01, 5A Engineering Drive 1, Singapore 117411, Singapore; 6 Singapore-MIT Alliance for Research and Technology, 1 CREATE Way, #10-01 CREATE Tower, Singapore 138602, Singapore; 7 NUS Graduate Programme in Bioengineering, NUS Graduate School for Integrative Sciences and Engineering, National University of Singapore, Singapore 117597, Singapore; 8 Department of Biological Engineering, Massachusetts Institute of Technology, Cambridge, MA 02139, USA; 9 Gastroenterology Department, Southern Medical University, Guangzhou 510515, China; 10 Division of Biotechnology, IFM, Linköping University, Linköping 58183, Sweden; 11 Department of New Biology, Daegu Gyeongbuk Institute of Science and Technology (DGIST), Daegu 42988, Korea

**Keywords:** 3D cell culture, microfluidics, cell migration, cell invasion, metastasis

## Abstract

We have developed a microfluidic-based culture chip to simulate cancer cell migration and invasion across the basement membrane. In this microfluidic chip, a 3D microenvironment is engineered to culture metastatic breast cancer cells (MX1) in a 3D tumor model. A chemo-attractant was incorporated to stimulate motility across the membrane. We validated the usefulness of the chip by tracking the motilities of the cancer cells in the system, showing them to be migrating or invading (akin to metastasis). It is shown that our system can monitor cell migration in real time, as compare to Boyden chambers, for example. Thus, the chip will be of interest to the drug-screening community as it can potentially be used to monitor the behavior of cancer cell motility, and, therefore, metastasis, in the presence of anti-cancer drugs.

## 1. Introduction

Metastasis is a leading cause of death in patients with malignant neoplasms [1]. The mechanism of metastasis has been under intense research and may translate into effective cancer therapies [2,3,4]. Cancer metastasis progresses in multiple steps. It involves the loss of cell adhesion from the primary tumor, increased cell motility and invasion across the basement membrane into the blood capillary (intravasation), systemic circulation and, finally, extravasation into surrounding tissues [5]. The assays for cancer metastatic potential are typically pursued in in vivo models since they have all the necessary cues essential for successful metastasis [6,7]. However, animal models are expensive and difficult to multiplex [8]. Moreover, it is difficult to isolate and study the multi-factorial processes contributing to metastasis. In vitro models allow for more controlled experimentation to better understand specific processes, such as migration and invasion, leading to cancer metastasis [9].

The development of microfluidic systems as in vitro cancer cell migration models, in particular, offers advantages over conventional methods of studying cancer cell migration and invasion, such as Boyden chambers [10,11,12] and scratch tests [12,13,14]. Microfluidic-based cancer migration models minimize the requirements for reagents and cells [15]. Such a platform is particularly useful for the study of small cancer cell populations, such as cancer stem cells or cells obtained from clinical patient specimens. Microfluidic cancer migration models also allow the application of chemo-attractant gradients [16,17], improve imaging resolution [18] and can look at interaction with other cells [19,20]. However, cancer cells in these microfluidic cancer migration models are cultured 2-dimensionally, rendering them only suitable for studying the inherent genetic migratory disposition of a cancer cell population. These models lack the context of a 3D tumor microenvironment to investigate the onset and progression of cancer cell migration and invasion, which has been increasingly implicated in cancer metastasis [8,21,22]. Improvements have been achieved by incorporating Matrigel^TM^ lining [18] or collagen scaffolds [23] into microfluidic cell migration models to observe how cancer cells migrate across a 3D barrier. One such a barrier was formed of an endothelial layer in which the intravasation of cancer cells was monitored [24]. It should also be noted that cancer cell density influences the cell migration [25,26]. Therefore, as cell migration starts with solid tumors, it is imperative to include solid cancer cell aggregates in the migration model to study cancer. This has not been the case in most of the previous studies.

Here, we describe a microfluidic cancer cell migration model that allows cancer cells to form 3D cellular aggregates resembling cancer tumors before initiating cancer cell migration and invasion. To mimic the basement membrane, a 3D collagen barrier is then formed around the 3D cancer cell aggregate via a polyelectrolyte complex coacervation process described by Toh et al. [27]. We were able to observe, in real time, the migration and invasion of a metastatic breast cancer cell (MX-1) from a 3D cellular aggregate across a collagen barrier. Our system also has excellent optical properties and allows multi-dimensional (x,y,z, time) acquisition of the cell migration and invasion process at high resolution. Thus, our microfluidic cancer cell migration model presents an opportunity to study cancer cell migration at high spatial and temporal resolution in a more biologically relevant 3D setting. 

## 2. Materials and Methods

**Figure 1:**
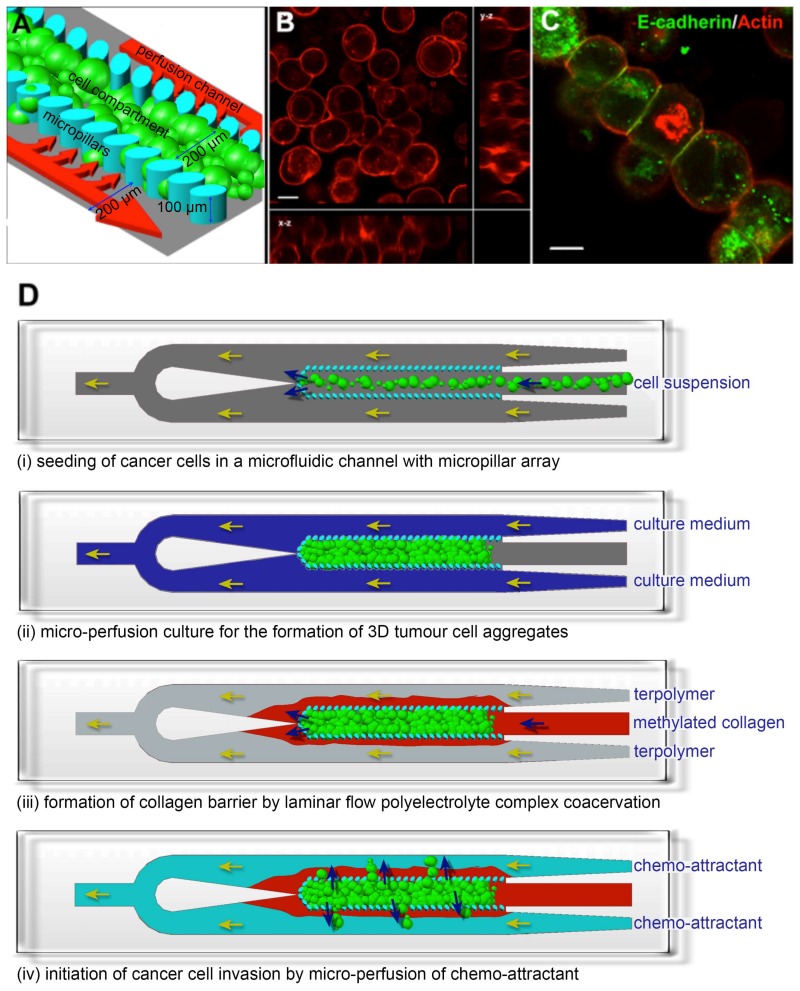
Establishment of a 3D cancer cell migration model in a microfluidic channel. (**A**) An array of 30 × 50 μm micropillars separated the microfluidic channel into 3 compartments: a central cell culture compartment and 2 side media perfusion compartments. Cancer cells are immobilized 3-dimensionally at high density within the central cell compartment and will remodel into 3D cellular aggregates after perfusion culture; (**B**,**C**) show the 3D cellular phenotype of MCF7, a breast cancer cell line, after 3 days of perfusion culture. (**B**) Rhodamine-phalloidin staining revealed cortical actin distribution. Image is an orthogonal projection of a 30 μm thick confocal optical section; (**C**) E-cadherin immunofluorescence staining confirmed the presence of cell-cell interactions within the 3D cellular aggregate. Scale bars = 10 μm; (**D**) Schematics for performing the cancer cell migration/invasion assay using the microfluidic model. (**i**) Cancer cells are seeded into the microfluidic channel and (**ii**) perfusion-cultured for 3 days to allow formation of 3D cellular aggregate. (**iii**) A collagen barrier is formed around the 3D cellular aggregate by laminar flow complex coacervation of a positively charged collagen and a negatively charged HEMA-MMA-MAA terpolymer. (**iv**) Cancer cell migration/invasion is then initiated by perfusing chemo-attractant through the side perfusion channels.

We first formed a 3D cancer aggregate by engineering a 3D microenvironment within a 1 cm (length) × 600 μm (width) × 100 μm (height) polydimethylsiloxane (PDMS) microfluidic channel. The fabrication of the microfluidic channel was previously described by Toh et al. [27]. An array of 30 × 50 μm elliptical micropillars with a gap size of 20 μm separated the microfluidic channel into 3 compartments: a 200 μm wide central cell culture compartment flanked by 2 side perfusion compartments. The pillar dimensions and gap size determine the porosity of the pillar array and, therefore, the exposure of the cells towards sheer stress of the perfused medium and the level of diffusion of nutrients and waste across the pillar array. The micropillar array within the microfluidic channel immobilizes cells at high density, forming 3D cell-cell interactions (Figure 1A). After the cells were seeded, a cell-conforming layer of 3D matrix was formed by the laminar flow complex coacervation of a positively-charged modified collagen and negatively-charged acrylate-based terpolymer to present the cells with 3D cell-matrix interactions [27]. Cancer cells cultured in this 3D microfluidic cell culture system remodeled into a 3D cellular aggregate, which exhibited cortical actin localization and expressed the cell adhesion protein E-cadherin (Figure 1B,C), indicating a high cell density with tight junctions. After the formation of a 3D cellular aggregate, we constructed a collagen barrier around the aggregate to simulate the basement membrane which migrating cancer cells must transverse during invasion (Figure 1D). The collagen barrier was formed by using the laminar flow complex coacervation process as described previously. However, the ratio of the negatively charged acrylate-based terpolymer to positively charged modified collagen was kept <5 to ensure a sufficiently thick collagen gel to be formed [27]. To induce cancer cells to migrate from the center cell compartment to the side channels, chemo-attractants were perfused through the two side channels (Figure 1D). Due to the slow diffusion rate of the chemo-attractant across the collagen barrier [28], a gradient was maintained for the duration of the experiments.

We validated our microfluidic cancer cell migration model with a breast cancer cell model. MX-1 cells were routinely maintained in RPMI medium (Invitrogen, Singapore) with 10% fetal calf serum (FCS) (Invitrogen, Singapore), 1.5 g/L sodium bicarbonate (Invitrogen, Singapore), 1 mM sodium pyruvate (Invitrogen, Singapore) and 1.5 g/L L-glutamate (Invitrogen, Singapore) at 37 °C, 5% CO_2_. The cells were seeded into the 3D microfluidic cell culture system at a density of 5 × 10^6^ cells/mL and perfusion-cultured at a flow rate of 0.03 mL/h for 3 days to allow formation of 3D MX-1 aggregates. During this period, the cells were serum-starved from 10% to 5% FCS, with a 2.5% reduction in serum concentration every 24 h. Upon formation of the 3D MX-1 aggregates, the collagen barrier was formed around the aggregate. The cell migration assay was initiated by perfusing a chemo-attractant (RMPI medium with 20% FCS and 60 mM HEPES (Invitrogen, Singapore)) through the side channels at a flow rate of 0.02 mL/h. The migration of MX-1 cells from the 3D aggregate across the collagen barrier into the side perfusion channels was captured by time-lapse video (Streampix version 3.12.2, NorPix Inc., Quebec, Montreal, Canada) on a heated stage at 37 °C of a microscope (Olympus, Tokyo, Japan) for 45 h. Two groups (migratory and invading) of 3 cells were tracked by marking their position in the time-lapse video. Their x,y coordinates were then determined in Mathematica (Wolfram Research Inc., Champaign, IL, USA). From the time-lapse video the number of invading cells and cells in the channel section were estimated which gave the percentage of invading cells in the system. The migration rate was calculated as the total distance travelled in 45 h and averaged over the 3 cells in each group. We compared the invasiveness of MX-1 cells in the microfluidic model with that seen in conventional Boyden chambers. For Boyden chamber cell invasion assay, 150,000 cells were seeded in each ECM coated invasion assay chamber (Cell Invasion Assay Kit, Merck Millipore, Darmstadt, Germany) with 5% serum and placed in 24 well plates containing media with 20% serum to act as a chemo-attractant for invasion. Wells were assayed every 12 h, where the cells and matrix inside the wells were removed using cotton swabs and the bottom of the well was immersed in a cell staining solution (Merck Millipore, Darmstadt, Germany) for 20 min, rinsed in water and the number of invasive cells were counted.

## 3. Results and Discussion

**Figure 2:**
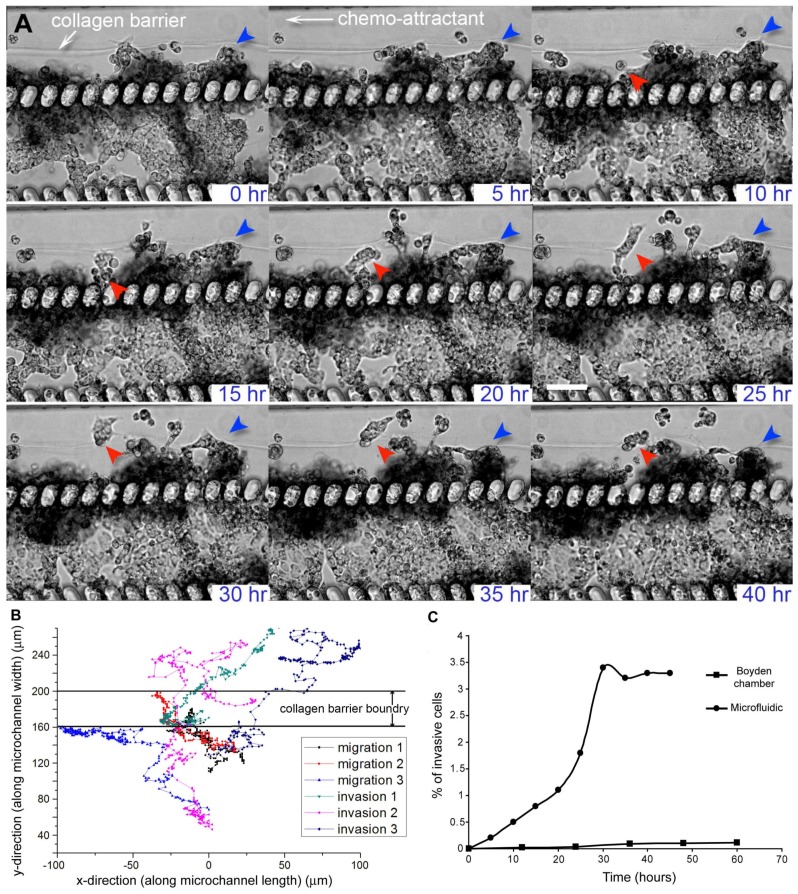
Migration and invasion of MX-1, a metastatic breast cancer cell line, in the microfluidic cancer cell migration model. (**A**) Time-lapse transmission images showing that MX-1 cells exhibited different modes of motility as they migrated or invaded across the collagen barrier over a period of 40 h. Most of the cells migrated collectively instead as single cell (red and blue arrows). Cells also exhibited plasticity in their mode of motility. Cells (in red arrow) displayed mesenchymal-like motility for up to 35 h before switching to amoeboid-like motility. Scale bar = 100 μm; (**B**) Migration trajectories of MX-1 cells showed the presence of 2 cell populations. Cells that transmigrated across the collagen barrier were defined as invading cells while cells that were motile but did not transmigrate across the collagen barrier were defined as migratory cells. The boundary of the collagen barrier varied over a range of 40 μm because migrating cells caused distention of the barrier at localized regions; (**C**) % of invasive MX-1 cells in the microfluidic cell migration model and control collagen-coated Boyden chamber.

In our microfluidic cancer cell migration model, we observed that MX-1 cells were able to invade across the collagen barrier into the side perfusion channels within 45 h (Figure 2A, Supplementary Video S1). The invading MX-1 cells in the microfluidic model exhibited both amoeboid-like motility, where the cells had amorphous cell morphology and changed direction rapidly and mesenchymal-like motility, where cells are elongated and form membrane protrusions at the leading edge [8,29] (Figure 2A). The amoeboid mode of cancer cell motility is usually only observed in animal or 3D in vitro models, and is mechanistically different from mesenchymal motility observed when cells are cultured 2-dimensionally [8,29]. We were also able to observe collective motility where cells retained their cell-cell contacts and invade as a group (Figure 2A). This mode of cancer cell motility has previously been observed in animal models only and is poorly understood because in vitro models have not been successful in modeling such motility [8,29]. The 3D tumor microenvironment in our microfluidic model provides the necessary cues to allow cancer cells to exhibit different modes of motility as compared to those typically observed in Boyden chambers and other 2D cell migration models. Since the underlying mechanisms for different modes of motility are different and there is plasticity in motility modes, anti-metastatic drugs that are successful in inhibiting mesenchymal cell migration in 2D cell migration models (e.g., Boyden chambers) may not be clinically effective [8,30]. Our system can function as an alternative or complementary testing model to existing cell migration models for evaluating a combination of drug inhibitors targeting different modes of cancer cell motility. 

While the majority of the metastatic MX-1 cells were highly motile when compared to non-metastatic breast cancer cell lines, such as MCF7 (data not shown), not all cells within the 3D tumor aggregate have equal tendency to invade. Also, other cancer cell lines, such as liver, kidney, lung, or beta cells, have not been observed to migrate within the presented system [27,31,32,33].

MX-1 cells that transmigrated across the collagen barrier were defined as invading cells while cells that were motile but did not transmigrate across the collagen barrier were defined as migratory cells. The presence of 2 distinct migrating and invading cell populations was more apparent when we tracked the trajectories of different cells (Figure 2B). The average velocities of the migrating and invading cells were 6.6 ± 1.5 μm/h and 13.5 ± 5.5 μm/h respectively. This observation supports the existence of a heterogeneous cell population within a tumor, where some cells have a higher tendency to intravasate into blood capillaries, thus increasing the likelihood of cancer metastasis [34]. Moreover, when we compared the percentage of cell invasion in the microfluidic model and collagen-coated Boyden chamber, we found that the percentage of invaded cells in the microfluidic model was approximately 10-fold higher than that of the Boyden chamber (Figure 2C). The number of invasive cells will stay constant after a stable gradient condition of the chemo-attractant has been reached. There is increasing evidence showing that a more invasive phenotype is a result of not only intrinsic genetic variation but also a different tumor microenvironment (i.e., ECM and surrounding cells) [5,34]. Cancer cell invasion assays that use in vitro cancer models where cells are cultured on 2D substrates are unable to model the tumor microenvironment adequately to account for the effect of the microenvironment on the invasiveness of cancer cells. In comparison, a 3D tumor microenvironment is represented in our microfluidic cancer invasion model by the construction of 3D tumor aggregate and 3D collagen matrix. Thus, our microfluidic model can facilitate experiments to understand the role of the tumor microenvironment in the development of heterogeneity and invasiveness of cancer cell phenotypes.

In vivo cell migration occurs dynamically in 3D space. To fully study this complex process, not only must an in vitro model recapitulate the 3D tumor microenvironment, it must also be able to capture this process in multiple dimensions (x,y,z, time) at high resolution [35]. The microfluidic cell migration model is designed such that the cellular phenotype and migration trajectory can be imaged and captured at high resolution. The imaging plane of our microfluidic model is perpendicular to the ECM barrier through which cancer cells transmigrate. This design facilitates high-resolution imaging of the migration process as compared to conventional Boyden chambers where the imaging plane is parallel to the ECM barrier [8,36]. In Figure 2, we demonstrated the use of transmission time-lapse microscopy to capture the migration of MX1 cells across the collagen barrier. The microfluidic cell migration model is also amenable to imaging by high-resolution imaging modalities such as laser scanning confocal microscopy, since it is bonded onto glass coverslips. This allows improved spatial resolution and the acquisition of 3D information. We demonstrate this by using laser scanning confocal microscopy to image fluorescent labeled MX-1 cells and collagen matrix in multi-dimensions (x,y,z, time). MX-1 cells were GFP-labeled while the methylated collagen used for forming the collagen barrier was labeled with Alexa Fluor 532 (Invitrogen, Waltham, MA USA) [27]. MX-1 cells were seeded into the microfluidic channel and initiated to migrate as described above. The microfluidic system was placed on a heated stage of a confocal microscope (Zeiss LSM, Jena, Germany) with chemo-attractant (RMPI with 20% FCS and 0.06 mM HEPES (Invitrogen, Singapore) being perfused constantly at a flow rate of 0.02 mL/h. An optical stack of 100 μm (dz = 10 μm) was imaged over 24 h.

**Figure 3:**
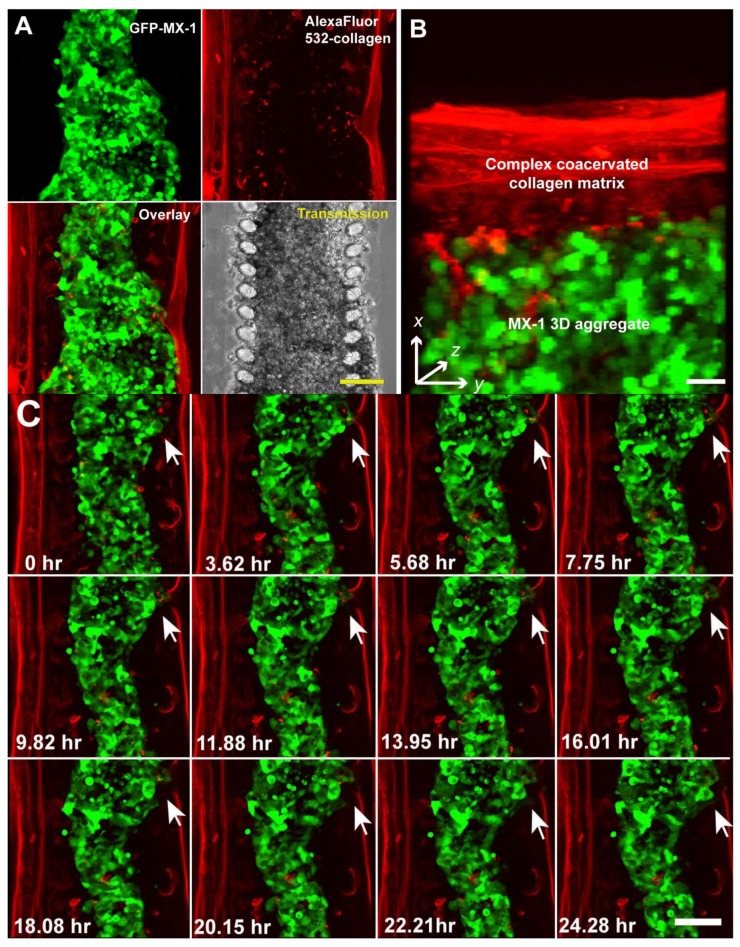
Multi-dimensional (x, y, z, time) imaging of cancer cell migration in the microfluidic cell migration model using a laser scanning confocal microscope. (**A**) MX-1 cells and collagen matrix were labeled with GFP and Alexa Fluor 532 respectively so that they can be imaged independently. Scale bar = 100 μm; (**B**) 3D reconstruction of a 10 μm optical section at 1 μm interval. Scale bar = 50 μm; (**C**) 24 h time-lapse confocal imaging showing remodeling of the collagen matrix by invading MX-1 cells (white arrow). Scale bar = 100 μm.

MX-1 cells and the collagen matrix in the microfluidic channel can be resolved more clearly by fluorescence confocal imaging than transmission imaging (Figure 3A). The 3D MX-1 aggregate was encapsulated by a fibrous collagen matrix approximately 50 μm thick. The thickness of this collagen barrier is comparable to that of commercially available ECM-coated membranes used in invasion assays with Boyden chambers. Compatibility of our microfluidic cell migration model with fluorescence imaging potentially allows different cell populations in a tumor aggregate, such as cancer-initiating and non-initiating cells [37], to be differentially labeled and tracked during the intravasation process. 3D information can be extracted by performing a 3D reconstruction of the model as shown in Figure 3B or by performing image processing of the individual sections of the optical stack. As observed using transmission microscopy, invasion of MX-1 cells across the collagen barrier occurred at localized regions (Figure 3C). Confocal microscopy revealed that invasion was initiated by the establishment of contact between migrating cells and the collagen matrix at a localized spot, followed by degradation of the collagen matrix over a period of 24 h (Figure 3C). Our microfluidic cancer migration model was able to clearly distinguish hallmark events of cancer intravasation (i.e., increased cell motility, diminished cell adhesions and proteolytic disruption of the ECM) [5] at real time in 3D space. When compared to models that assay for end points such as number of cells migrated [18,38], our microfluidic model shows more versatility for screening anti-metastatic drugs specifically targeting a process of intravasation. For example, we can evaluate the efficacy of anti-metastatic drugs targeting at metalloproteinases (MMPs) [39] by tracking the degradation of the collagen matrix over time. Alternatively, measuring changes in the rate of cell migration can help to evaluate the effectiveness of drugs targeting cell motility [40].

## 4. Conclusions

In conclusion, we developed a microfluidic cell migration model that can resolve different aspects of cancer cell intravasation (i.e., loss of cell adhesion, different modes of cell motility and ECM degradation) at high resolution in a biologically relevant 3D environment. We were able to accomplish this by incorporating a 3D microenvironment, which has been previously shown to affect the invasiveness of cancer cells, into a transparent microfluidic system. This microfluidic cancer cell migration model not only has high imaging resolution and resemblance to the in vivo situation, but it is also amenable to multiplexing to achieve high-throughput assaying. Hence, our microfluidic cancer cell migration model is appealing for anti-metastasis drug testing, especially drugs targeting migration and invasion.

## Supplementary Materials

The following are available online at http://www.mdpi.com/2306-5354/5/2/29/s1, Video S1: MX1_0_48hr.avi.
